# Systemic energy metabolism changes in patients with heart failure receiving specific oral nutritional supplements in combination with Mediterranean diet

**DOI:** 10.3389/fnut.2026.1880396

**Published:** 2026-08-05

**Authors:** Aura D. Herrera-Martínez, Concepción Muñoz Jiménez, José López Aguilera, Manuel Crespin, María Ángeles Gálvez Moreno, María José Molina Puerta

**Affiliations:** 1 Maimonides Institute for Biomedical Research of Cordoba (IMIBIC), Córdoba, Spain; 2 Endocrinology and Nutrition Service, Reina Sofia University Hospital, Córdoba, Spain; 3 Cardiology Service, Reina Sofía University Hospital, Córdoba, Spain

**Keywords:** heart failure, isoleucine, nutritional support, oral nutritional supplement (ONS), valine

## Abstract

**Background:**

Heart failure (HF) is associated with disease-related malnutrition, all of which contribute to impaired functional capacity and adverse outcomes. Nutritional strategies combining Mediterranean diet (MD) patterns with oral nutritional supplements (ONS) have shown clinical and anti-inflammatory benefits in HF.

**Objectives:**

To evaluate the impact of MD combined with hypercaloric, hyperproteic, omega-3–enriched ONS vs. MD alone on targeted metabolomic profiles related to energy metabolism, skeletal muscle metabolism, and amino acid handling in patients with HF.

**Methods:**

This study is a secondary analysis of a prospective, randomized, open-label clinical trial including patients with chronic HF. Participants were randomized to receive either MD alone or MD plus two hypercaloric, hyperproteic, omega-3–enriched ONS for 24 weeks. Targeted metabolomic profiling was performed using proton nuclear magnetic resonance, assessing glycoproteins, energy metabolism–related metabolites, muscle-related metabolites, and amino acid metabolism–related metabolites.

**Results:**

Thirty-eight patients were included, of whom 65.8% presented sarcopenia. Glycoprotein fraction levels significantly decreased over time in the whole cohort, without differences between intervention groups. In energy metabolism–related parameters, glucose and pyruvate significantly decreased in the DM–only group, whereas glycerol levels showed a trend toward increase in the intervention group, reaching statistical significance in patients without sarcopenia. Regarding muscle-related metabolites, leucine levels significantly increased in the intervention group, while isoleucine decreased in the control group (*p* < 0.05). In patients with sarcopenia, the intervention was associated with increases in valine and leucine (*p* < 0.05), whereas no significant muscle-related metabolomic changes were observed in non-sarcopenic patients. In amino acid metabolism, threonine decreased in the control group, while glycine significantly increased in sarcopenic patients receiving combined nutritional support (*p* < 0.05). Baseline NT-proBNP levels positively correlated with inflammatory glycoproteins and inversely correlated with glycine and proline. Multivariate analysis showed that baseline isoleucine levels were independently associated with mortality after adjustment for age, sex, type 2 diabetes, NT-proBNP levels, left ventricular ejection fraction, and body mass index, baseline isoleucine and valine remained independently associated with mortality (OR 0.77, 95% CI 0.591–0.9998; OR 0.95, 95% CI 0.907–0.999).

**Conclusions:**

In patients with HF, nutritional support combining MD with hypercaloric, hyperproteic, omega-3–enriched ONS was associated with distinct metabolomic adaptations, particularly affecting branched-chain amino acid profiles in patients with sarcopenia. These findings suggest a potential role for comprehensive nutritional strategies in modulating systemic and muscle-related metabolic pathways linked to functional status and prognosis in high-risk HF populations.

## Introduction

Heart failure (HF) is characterized not only by impaired cardiac pump function but also by profound systemic metabolic disturbances that extend beyond the myocardium to involve skeletal muscle mass, muscle bioenergetics, and whole-body energy homeostasis (1). In recent years, HF has increasingly been conceptualized as a state of systemic metabolic dysregulation, in which impaired substrate utilization and reduced energetic efficiency interact with inflammation, neurohormonal activation, and nutritional deficiencies to accelerate disease progression (1, 2).

A central feature of HF-related metabolic dysfunction is the loss of metabolic flexibility (2, 3). Such metabolic inflexibility amplifies oxidative stress and contributes to progressive ventricular dysfunction, thereby increasing the risk of decompensation and hospitalization (2). Circulating intermediates such as glucose, lactate, pyruvate, acetoacetate, and glycerol reflect the integrated metabolic state of cardiac and peripheral tissues and have emerged as accessible biomarkers of altered oxidative metabolism and substrate utilization in HF (2).

Skeletal muscle represents a critical peripheral target of HF-related metabolic derangements, which limit oxygen delivery and ATP resynthesis during exertion, exacerbating functional limitation independently of cardiac function (4). In parallel, enhanced proteolysis and reduced protein synthesis promote progressive muscle wasting and sarcopenia, which are strongly associated with reduced strength, lower peak oxygen consumption, diminished quality of life, and poorer survival (1). The metabolic footprint of HF-related skeletal muscle dysfunction is reflected in circulating levels of muscle-related metabolites, including creatinine, creatine, tyrosine, and branched-chain amino acids (BCAAs; valine, leucine, and isoleucine) (1).

Sarcopenia and sarcopenic obesity are highly prevalent across the spectrum of HF phenotypes, including both reduced and preserved ejection fraction, reflecting the combined effects of chronic inflammation, neurohormonal activation, physical inactivity, and inadequate nutrient intake (1, 5). Furthermore, disease-related malnutrition is common in chronic HF, particularly among patients with recent hospitalizations (6); in this context, hypercaloric, hyperproteic oral nutritional supplements (ONS) are recommended to correct negative energy balance, support anabolic pathways, and counteract muscle wasting in malnourished or high-risk patients with chronic disease, including HF (2). Randomized and pragmatic studies in HF and related populations have shown that high-energy, high-protein ONS can improve body weight, nutritional biomarkers, muscle strength, and functional performance (6).

Nutritional interventions have gained increasing attention as modulators of metabolic dysfunction in HF; the Mediterranean diet has emerged as a cardioprotective dietary pattern associated with reduced cardiovascular morbidity and mortality, improved metabolic health, and lower incidence of HF (7). Several components, such as monounsaturated fatty acids, omega-3 polyunsaturated fatty acids (PUFAs), polyphenols, and antioxidants, target key mechanisms underlying HF-related metabolic dysfunction, including insulin resistance, inflammation, and mitochondrial impairment (2). Although Mediterranean-style dietary interventions have been associated with improvements in functional status and health-related quality of life, detailed mechanistic data linking this dietary pattern to systemic and muscle-specific metabolic adaptations in HF remain limited (7).

In a recent randomized clinical trial, nutritional support with hypercaloric, hyperproteic, omega-3–enriched ONS added to a Mediterranean diet resulted in significant reductions in inflammatory cytokines and improvements in cardiac biomarkers compared with Mediterranean diet alone (5, 8). However, the metabolic pathways underlying these clinical benefits, particularly those related to systemic energy metabolism and skeletal muscle, remain incompletely elucidated (8).

Targeted metabolomics provides a clinically relevant approach to characterize specific metabolic adaptations induced by nutritional interventions in HF (2). The present study addresses this knowledge gap by performing a secondary targeted metabolomic analysis within a randomized clinical trial comparing Mediterranean diet alone vs. Mediterranean diet combined with hypercaloric, hyperproteic, omega-3–enriched ONS in patients with chronic HF and at least one hospitalization in the preceding 6 months (8). The objective is to characterize changes in selected energy metabolism–related, muscle-related, and amino acid–related metabolites, thereby providing novel mechanistic insights into how comprehensive nutritional support may modulate systemic metabolic flexibility, skeletal muscle metabolism, and functional potential in this high-risk HF population (8).

## Material and methods

### Patients

This study was approved by the Ethics Committee of Reina Sofía University Hospital (Córdoba, Spain; reference number 5164), initially authorized on October 21, 2021, and subsequently updated on May 30, 2023. The study was conducted in accordance with the Declaration of Helsinki and complied with national and international ethical guidelines. A prospective, open-label design was used, and written informed consent was obtained from all participants prior to inclusion. All individuals received detailed information about the study, and only those who agreed to participate were enrolled.

The cohort was originally recruited as part of an open, randomized, controlled clinical trial (ClinicalTrials.gov identifier: NCT05848960)(5). Eligible participants were men and women aged >18 and < 85 years, with a left ventricular ejection fraction (LVEF) < 50% and at least one hospital admission for heart failure within the previous 6 months. N-terminal pro–brain natriuretic peptide (NT-proBNP) levels were measured at baseline and at the end of the study. LVEF was assessed by transthoracic echocardiography at the same time points.

### Nutritional support

Within the clinical trial, participants were randomly assigned in a 1:1 ratio to receive either a Mediterranean diet alone or a Mediterranean diet combined with two daily servings of a hypercaloric, hyperproteic oral nutritional supplement (ONS) over a 24-week period. The ONS formulation included slow-release carbohydrates, a fiber blend, and a combination of n-3 and n-6 fatty acids, providing 385 mg of EPA and DHA per 100 mL. The supplements were supplied by Nutrisens^®^. Only patients with a treatment adherence of at least 75% were included in the analysis. Adherence to Mediterranean Diet was assessed using the MEDAS14 questionary, all patients reported a score >9 at the end of the study.

At baseline, all participants received standardized education and counseling on nutritional support, adherence to the Mediterranean diet, and physical activity. In addition, all patients received oral calcifediol supplementation. Nineteen patients were initially allocated to each study arm. Due to mortality during follow-up, fifteen patients completed the study in the control group and eighteen in the intervention group. Participants were further classified according to the presence or absence of sarcopenia (no sarcopenia, *n* = 13; sarcopenia, *n* = 25), defined as a handgrip strength below the 3rd percentile adjusted for age and sex. No other parameters were included for the definition of sarcopenia, since body composition analysis might be altered due to water content in these patients and other functional tests can be altered due to dyspnea, which is directly associated with HF.

### Glycoprotein analysis

Frozen samples were shipped to Biosfer Teslab (Reus, Spain) for NMR analysis. Acute-phase glycoproteins Glyc-A and Glyc-B were quantified using proton nuclear magnetic resonance (^1^H-NMR), as previously described (9). Low molecular weight metabolites (LMWMs) were determined, specifically energy metabolism-related parameters (glucose, lactate, pyruvate, acetone acetate, and glycerol); muscle-related parameters (creatinine, creatine, tyrosine, valine, isoleucine, and leucine); amino acid metabolism-related parameters (alanine, glutamate, glutamine, histidine, proline, and threonine).

### Statistical analysis

Between-group comparisons were analyzed by the Mann–Whitney U test (nonparametric data). Paired analysis was performed by Wilcoxon test (nonparametric data). Comparisons between more than two groups were performed using ANOVA. Chi-squared test was used to compare categorical data. *P*-values < 0.05 were considered statistically significant. Due to the small sample size, and to correct for multiple testing across variables, *p*-values were adjusted using the Holm–Bonferroni method. Statistical analyses were performed using SPSS statistical software version 20, and Graph Pad Prism version 6. Data are expressed as median with interquartile range and percentages. Absolute differences in some parameters were calculated using mean values. For specific group analysis, the absolute number has been also expressed in brackets.

## Results

Thirty-eight patients were included in this clinical trial, 65, 78% had sarcopenia (*n* = 25). Changes in metabolites were analyzed depending on the presence of sarcopenia; in this section only, significant results are present. Baseline clinical characteristics were similar in both groups but age, which was significantly higher in the group of sarcopenia (median 72 vs. 64, *p* = 0.02). General characteristics of the cohort is described in Table 1. Additionally, there were no statistically significant differences between the absolute levels of the analyzed metabolic compounds at baseline between patients with and without sarcopenia, but serum levels of valine and alanine tended to decrease in patients with sarcopenia (*p* = 0.06 and 0.05 respectively; Table 2).

**Table 1:** Baseline clinical characteristics of the patients.

Characteristics	Total (*n* = 38)	No sarcopenia (*n* = 13)	Sarcopenia (*n* = 25)	*P*
Sex (♂/♀)	71.1%/28.9% (11/27)	61.5%/38.5% (8/5)	24//76% (6/19)	0.29
Age (years)	66.7 ± 13	64 (20.5)	72.5 (22)	0.02
Active tobacco exposure (%)	42.10 (7/38)	46.2 (6/13)	40 (10/25)	0.49
Type 2 diabetes (%)	42.1 (16/38)	23.1 (3/13)	52 (13/25)	0.09
Previous ischaemic cardiomyopathy (%)	34.2 (13/38)	15.4 (2/11)	44 (11/25)	0.08
Ejection fraction (%)	38.5 ± 16	31.5 (30.25)	33.5 (21.5)	0.10
NT-proBNP (pg/ml)	5768 ± 6646	4616 (7202)	2692.5 (2990)	0.99
Symptoms				
Weight loss (3months, %)	55.3 (21/38)	46.2 (6/13)	60 (15/25)	0.32
Weight loss (6 months, %)	28.9	15.4 (2/13)	36 (9/25)	0.17
*Food intake* (%)				0.27
Soft	7.9 (3/38)	0	12 (3/25)	
Normal	92.1 (35/38)	100 (13/13)	88 (22/25)	
*Gastrointestinal symptoms* (%)	15.8 (6/38)	15.4 (2/13)	16 (4/25)	0.67
Abdominal pain	10.5 (4/38)	7.7 (1/13)	12 (3/25)	0.58
Nauseas/vomits	5.3 (2/38)	0	8 (2/25)	0.43
Diarrhea	5.3 (2/38)	7.7 (1/13)	4 (1/25)	0.57
Physical activity (%)				
Moderate	18.4 (7/38)	23.1 (3/13)	16 (4/25)	0.45
Resting time (hours/day)	8 ± 4	5 (6.25)	6.5 (5.5)	0.15
Quality of life				
Self-rated health score	69 ± 22	77.5 (18.75)	70 (31.25)	0.15
*Mortality*	13.16 (5/38)	7.69 (1/13)	16 (4/25)	0.43

Comparison between groups based on the presence of sarcopenia.

**Table 2:** Absolute levels of metabolomic compounds at baseline.

Characteristics	Total (n = 38)	No sarcopenia (*n* = 13)	Sarcopenia (*n* = 25)	*P*
Glycoproteins				
Glyc B	319.17 (88.77)	266.47 (108.10)	336.46 (75.50)	0.41
Glyc F	188.73 (59.55)	195.02 (65.96)	188.73 (60.37)	0.27
Glyc A	570.58 (158.38)	544.71 (238.09)	589.75 (151.15)	0.18
H W Glyc B	4.01 (1.12)	3.35 (1.36)	4.23 (0.95)	0.79
H W Glyc A	15.07 (4.40)	13.52 (5.66)	15.96 (3.46)	0.36
Muscle-related parameters				
Tyrosine	46.61 (21.42)	39.98 (36.14)	49.77 (17.58)	0.95
Valine	176.23 (65.20)	217.39 (59.39)	172.09 (49.77)	0.06
Isoleucine	45.03 (17.15)	59.98 (37.33)	41.57 (14.10)	0.15
Leucine	88.67 (41.37)	122.298 (51.70)	82.95 (38.63)	0.06
Creatinine	59.21 (19.93)	69.69 (23.31)	54.47 (15.42)	0.27
Creatine	71.84 (30.47)	87.75 (53.44)	67.20 (28.74)	0.22
Metabolism-related parameters				
Glucose	5.55 (3.14)	6.31 (4.06)	5.55 (2.76)	0.69
Lactate	934.66 (710.51)	773.81 (465.83)	999.52 (826.50)	0.10
Pyruvate	49.05 (25.41)	43.15 (24.40)	58.24 (25.60)	0.47
3-hydroxybutyrate	22.34 (7.65)	9.30 (27.93)	22.85 (28.92)	0.65
Acetate	5.45 (7.65)	11.49 (26.82)	5.44 (5.54)	0.19
Acetone	19.85 (129.41)	15.58 (10.09)	21.45 (20.22)	0.76
Glycerol	53.21 (37.44)	48.93 (29.81)	53.21 (58.36)	0.56
Amino acid metabolism-related parameters				
Alanine	392.91 (129.41)	439.55 (49.55)	383.44 (144.39)	0.05
Glutamate	287.40 (135.82)	298.11 (104.39)	287.40 (177.80)	0.27
Glutamine	257.51 (100.27)	282.76 (103.27)	240.54 (108.84)	0.83
Glycine	155.38 (49.97)	171.08 (38.79)	152.41 (56.62)	0.48
Histidine	44.57 (26.94)	56.36 (30.66)	42.82 (26.26)	0.54
Proline	139.71 (80.77)	136.75 (91.36)	145.96 (82.70)	0.90
Threonine	182.47 (57.93)	182.70 (33.37)	176.47 (65.76)	0.39

Comparison between groups based on the presence of sarcopenia. H-W, height to- Width ratio.

### Glycoproteins

**Figure 1:**
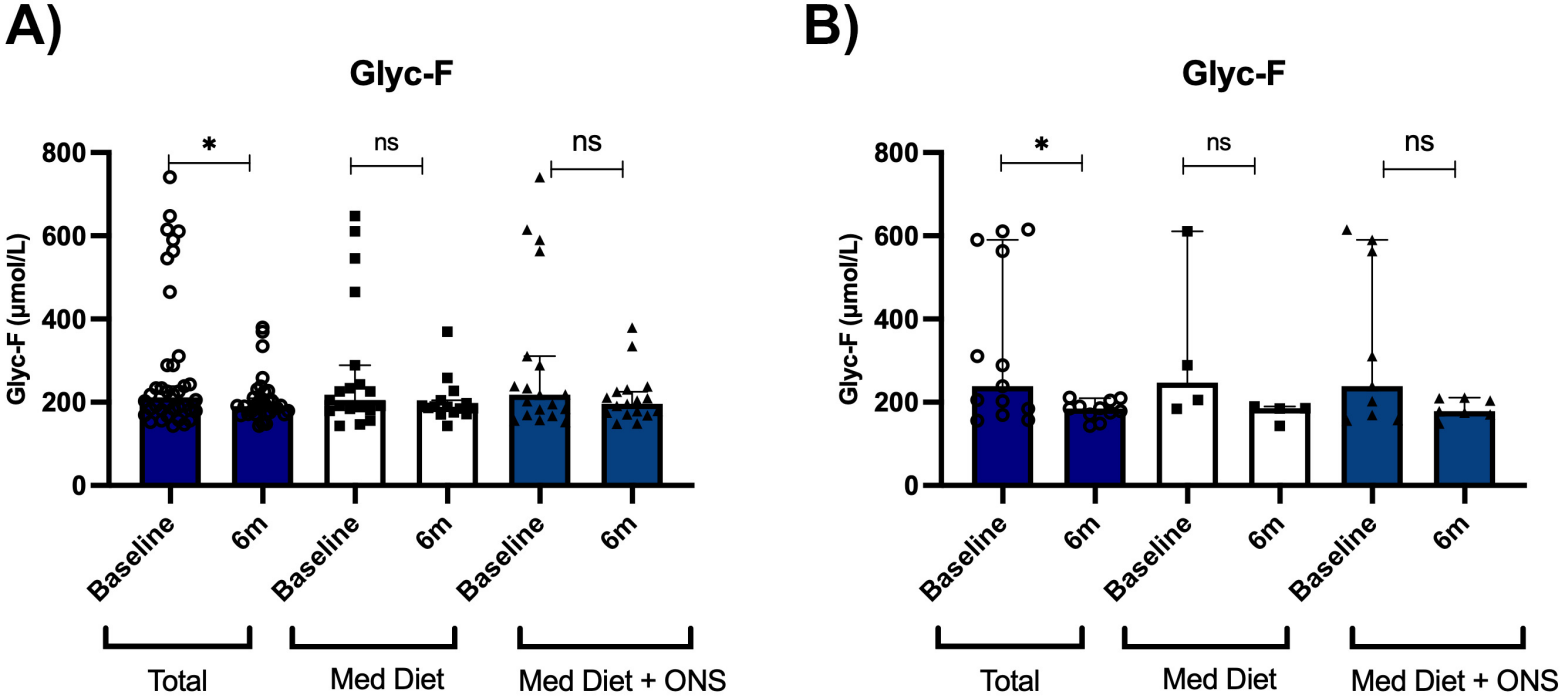
Significant metabolomic alterations in glycoprotein levels across control and intervention cohorts. Comparative analysis in the total study population **(A)** and in patients without sarcopenia **(B)**. Legend: Glyc-F, glycoprotein fraction; ns, non-significant; *p < 0.05.

Glycoprotein fraction (Glyc-F) significantly decreased in the whole cohort (*p* < 0.05; Table 3) but it significantly decreased in patients without sarcopenia (*p* < 0.05) while no significant changes were observed in patients with sarcopenia (Table 4). In the subgroup analysis, no significant differences between the control and intervention groups were observed (Figure 1A). No significant changes were observed in patients with sarcopenia. In patients without sarcopenia, Glyc-F also significantly decreased in the whole cohort (*p* < 0.05), without group-specific differences (Figure 1B).

**Table 3:** Metabolomic changes in the total cohort patients with heart failure after 6 months of nutritional support.

Characteristics	Baseline	6 months	*P*
Glycoproteins			
Glyc B	301.41 (94.57)	355.79 (65.96)	0.30
Glyc F	183.79 (63.65)	201.35 (75.10)	0.04
Glyc A	560.35 (179.96)	685.67 (210.10)	0.16
Muscle-related parameters			
Tyrosine	45.21 (17.94)	52.66 (91.36)	0.92
Valine	174.16 (91.36)	199.35 (71.78)	0.85
Isoleucine	42.87 (14.23)	37.24 (34.78)	0.49
Leucine Creatinine Creatine	90.24 (61.42)	88.80 (44.67)	0.96
Metabolism-related parameters			
Glucose	5.5 (4.69)	6.14 (4.35)	0.33
Lactate	1044.51 (683.16)	1062 (1178.90)	0.80
Pyruvate	53.57 (34.94)	55.39 (15.15)	0.14
Acetate	6.11 (9.75)	5.88 (4.86)	0.47
Acetone	22 (15.48)	23.94 (20.84)	0.40
Glycerol	54.80 (53.30)	67.23 (116.07)	0.25
3-hydroxybutyrate	22.60 (36.14)	25.76 (39.48)	0.98
Amino acid metabolism-related parameters			
Alanine	401.12 (240.25)	423.52 (411.87)	0.72
Glutamate	266.5 (168.06)	234.86 (77.49)	0.79
Glutamine	263 (80.54)	278.98 (80.54)	0.78
Glycine	157.85 (51.91)	167.08 (86.63)	0.14
Histidine	44.69 (29.51)	43.94 (18.92)	0.22
Proline	146.63 (88.82)	125.97 (75.23)	0.09
Threonine	170.09 (42.03)	200.76 (72.23)	0.91

Comparison between groups based on the presence of sarcopenia.

**Table 4:** Metabolomic changes in the total cohort patients with heart failure after 6 months of nutritional support.

Characteristics	No sarcopenia			Sarcopenia		
	Baseline	6 months	*P*	Baseline	6 months	*P*
Glycoproteins						
Glyc B	258.69 (94.57)	331.52 (65.96)	0.13	318.29 (99.38)	372.21 (71.94)	0.69
Glyc F	171.17 (63.65)	177.13 (26.89)	0.01	215.85 (67.93)	201.37 (121.34)	0.50
Glyc A	502.52 (179.96)	603.7 (145.5)	0.29	562.22 (172.96)	710.18 (223.06)	0.38
Muscle-related parameters						
Tyrosine	31.57 (5.74)	67.68 (19.74)	0.21	46.28 (14.77)	51.72 (10.13)	0.46
Valine	217.39 (7.82)	182.54 (62.64)	0.13	166.68 (98.52)	199.35 (78.64)	0.23
Isoleucine	56.55 (24.76)	33.85 (18.69)	0.21	39.10 (11.58)	37.37 (45.09)	0.94
Leucine	113.50 (31.92)	73.85 (18.14)	0.16	78.53 (58.25)	98.20 (59.19)	0.24
Creatinine	64.39 (15.31)	63.10 (16.56)	0.21	52.25 (10.89)	50.10 (22.82)	0.09
Creatine	69.87 (62.71)	78.74 (65.26)	0.42	72.60 (21.34)	73.27 (22.26)	0.90
Metabolism-related parameters						
Glucose	6.31 (2.75)	4.52 (2.43)	0.72	5.05 (5.12)	7.28 (4.72)	0.34
Lactate	773.81 (98.9)	1,369.91 (781.39)	0.85	1,225.50 (704.84)	1,062.18 (1,137.81)	0.52
Pyruvate	63.50 (16.45)	182.70 (16.45)	0.92	59.60 (29.14)	53.81 (11.57)	0.12
Acetate	21.82 (30.11)	4.23 (4.99)	0.26	4.90 (8.60)	7.23 (6.14)	0.88
Acetone	21.65 (12.33)	26.50 (20.61)	0.86	22.00 (19.36)	19.64 (28.61)	0.43
Glycerol	54.80 (53.30)	118.38 (122.76)	0.02	50.39 (75.16)	66.72 (102.11)	0.93
3-hydroxybutyrate	22.57 (32.52)	25.76 (39.48)	0.60	22.60 (44.21)	25.76 (53.16)	0.75
Amino acid metabolism-related parameters						
Alanine	446.63 (31.98)	458.45 (100.92)	0.53	376.80 (290.36)	393.84 (171.66)	0.98
Glutamate	305.98 (66.46)	300.37 (128.48)	0.59	218.42 (170.72)	233.04 (52.76)	0.93
Glutamine	259.83 (17.32)	260.29 (111.67)	0.06	273.25 (161.82)	278.08 (64.39)	0.38
Glycine	156.45 (7.74)	142.67 (45.60)	0.59	166.38 (98.35)	175.56 (98.35)	0.03
Histidine	56.36 (4.84)	31.59 (3.03)	0.04	38.66 (28.83)	49.76 (17.00)	0.63
Proline	184.98 (90.54)	154.16 (85.22)	0.01	146.63 (71.19)	125.97 (62.90)	0.74
Threonine	182.70 (46.03)	157.66 (7.77)	0.92	162.06 (76.949)	210.24 (86.29)	0.93

Comparison between groups based on the presence of sarcopenia.

### Energy metabolism-related parameters

There were no significant changes in the whole cohort when energy metabolism-related parameters were compared at baseline and after 6 months of nutritional support (Table 3). Glycerol significantly increased in patients without sarcopenia (*p* < 0.05) while no significant changes were observed in patients with sarcopenia (Table 4).

**Figure 2:**
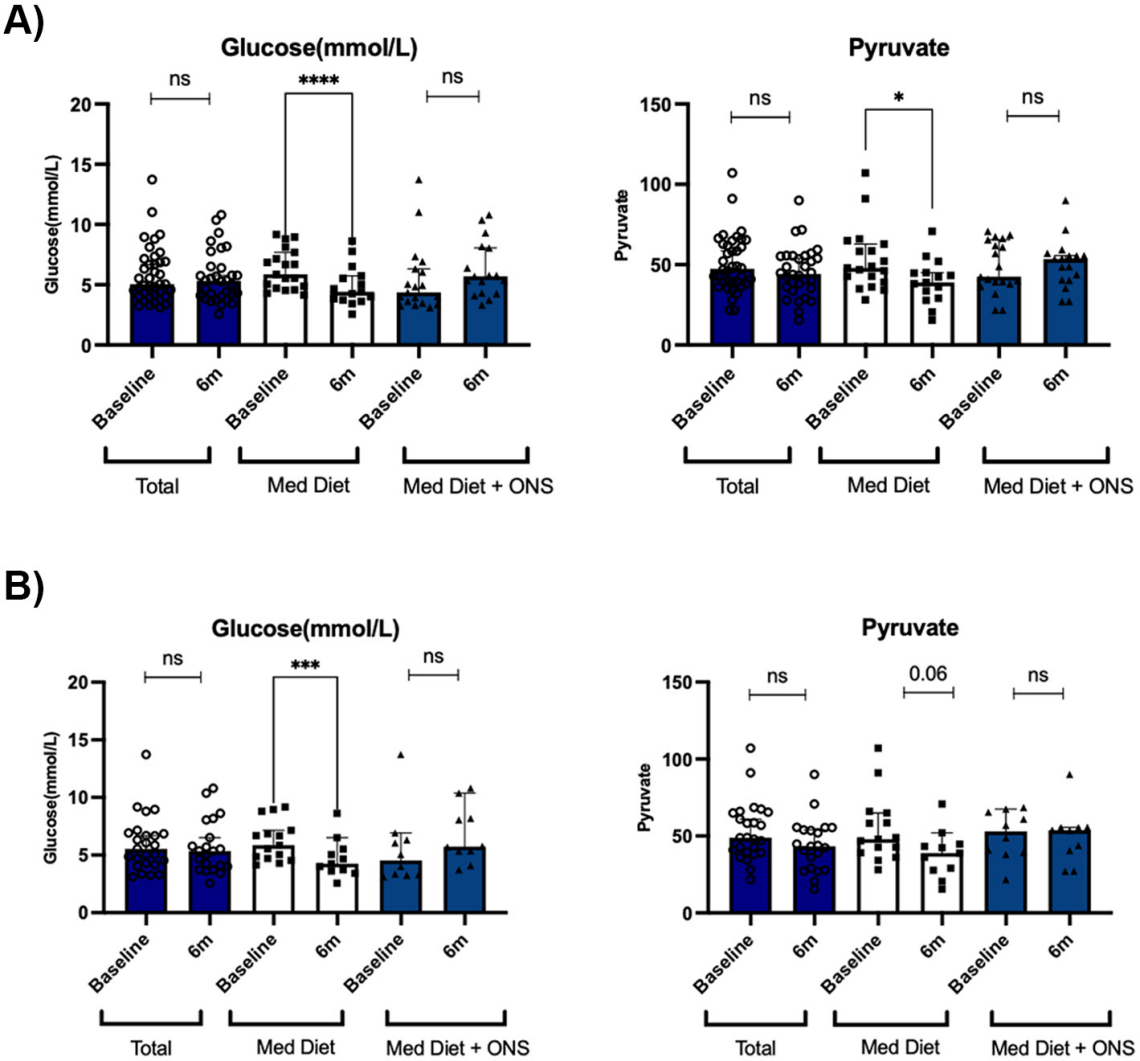
Significant shifts in energy metabolism-related metabolomic parameters. Data represent the total study population **(A)** and patients with sarcopenia **(B)**. Legend: ns, non- significant; *p < 0.05; ***p < 0.001; ****p < 0.0001.

At the cohort level, glucose and pyruvate levels significantly decreased in the control group (*p* < 0.0001 and *p* < 0.05, respectively), with no significant changes in the intervention group (Figure 2A).

In patients with sarcopenia, glucose levels significantly decreased in the control group (*p* < 0.001), while no significant changes were observed in the intervention group. Pyruvate showed a trend toward reduction in the control group (*p* = 0.06). No other significant changes were observed (Figure 2B).

### Muscle-related metabolism parameters

There were no significant changes in the whole cohort when muscle metabolism-related parameters were compared at baseline and after 6 months of nutritional support (Table 3).

**Figure 3:**
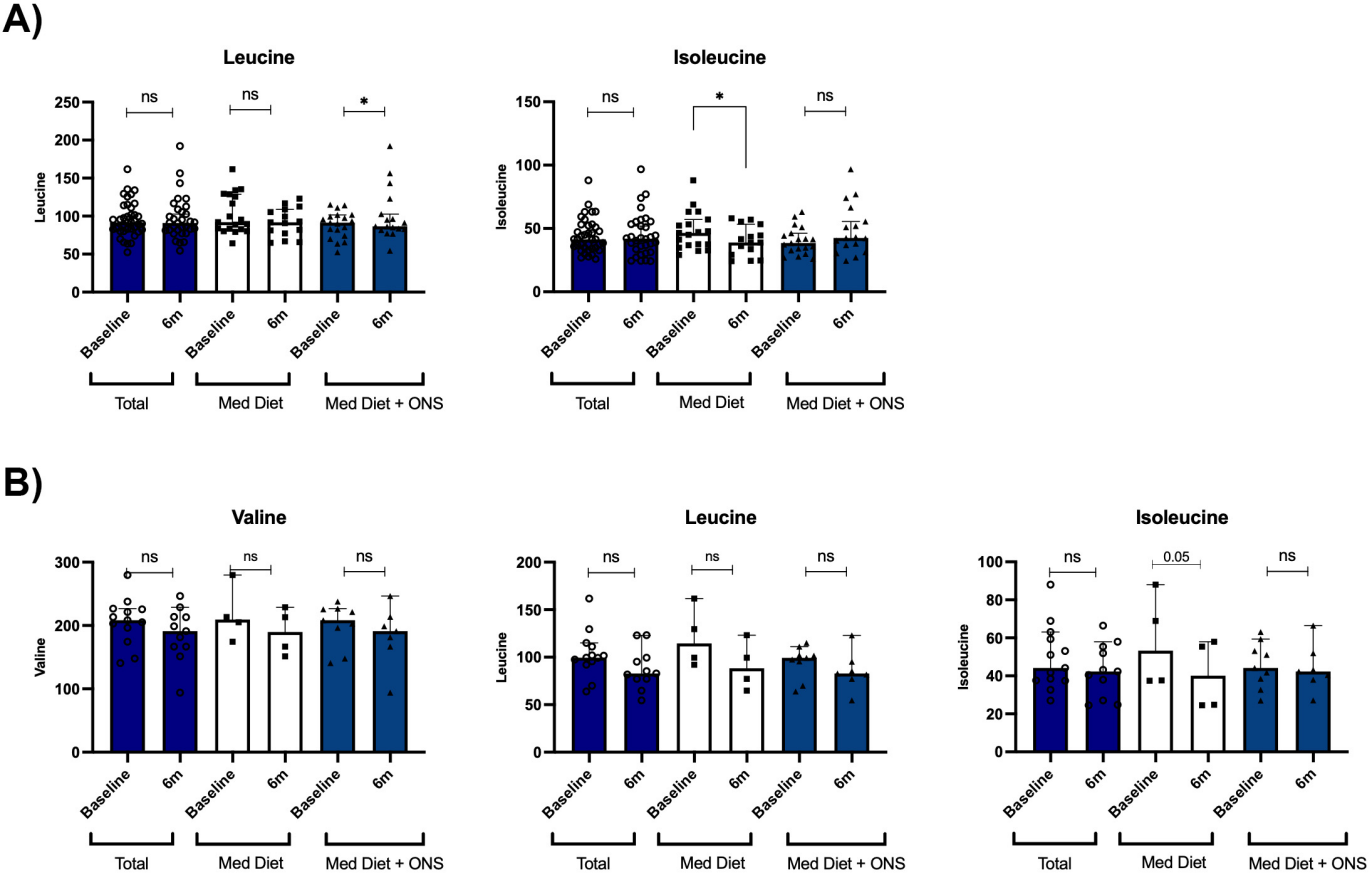
Significant metabolomic alterations in muscle-related metabolic parameters. Changes observed in the total study population **(A)** and in patients without sarcopenia **(B)**. Legend: ns, non-significant; *p < 0.05; **p < 0.01.

In the subgroup analysis, across the whole study population, leucine levels significantly increased in the intervention group (*p* < 0.05), whereas isoleucine significantly decreased in the control group (*p* < 0.05), with no changes in the intervention group (Figure 3A).

Among patients with sarcopenia, valine and leucine levels significantly increased in the intervention group (*p* < 0.01 and *p* < 0.05, respectively), while remaining unchanged in the control group. Isoleucine showed a trend toward decrease in the control group (*p* = 0.06). No other significant changes were observed (Figure 3B).

In patients without sarcopenia, no significant changes were observed in muscle-related metabolic parameters.

### Aminoacid metabolism

Regarding aminoacidic metabolism-related parameters, in the whole cohort, no significant changes were observed when baseline and 6 months values were compared (Table 3). In contrast, in patients without sarcopenia, histidine and proline levels significantly decreased (*p* < 0.05; Table 4). These changes were not observed in patients with sarcopenia.

**Figure 4:**
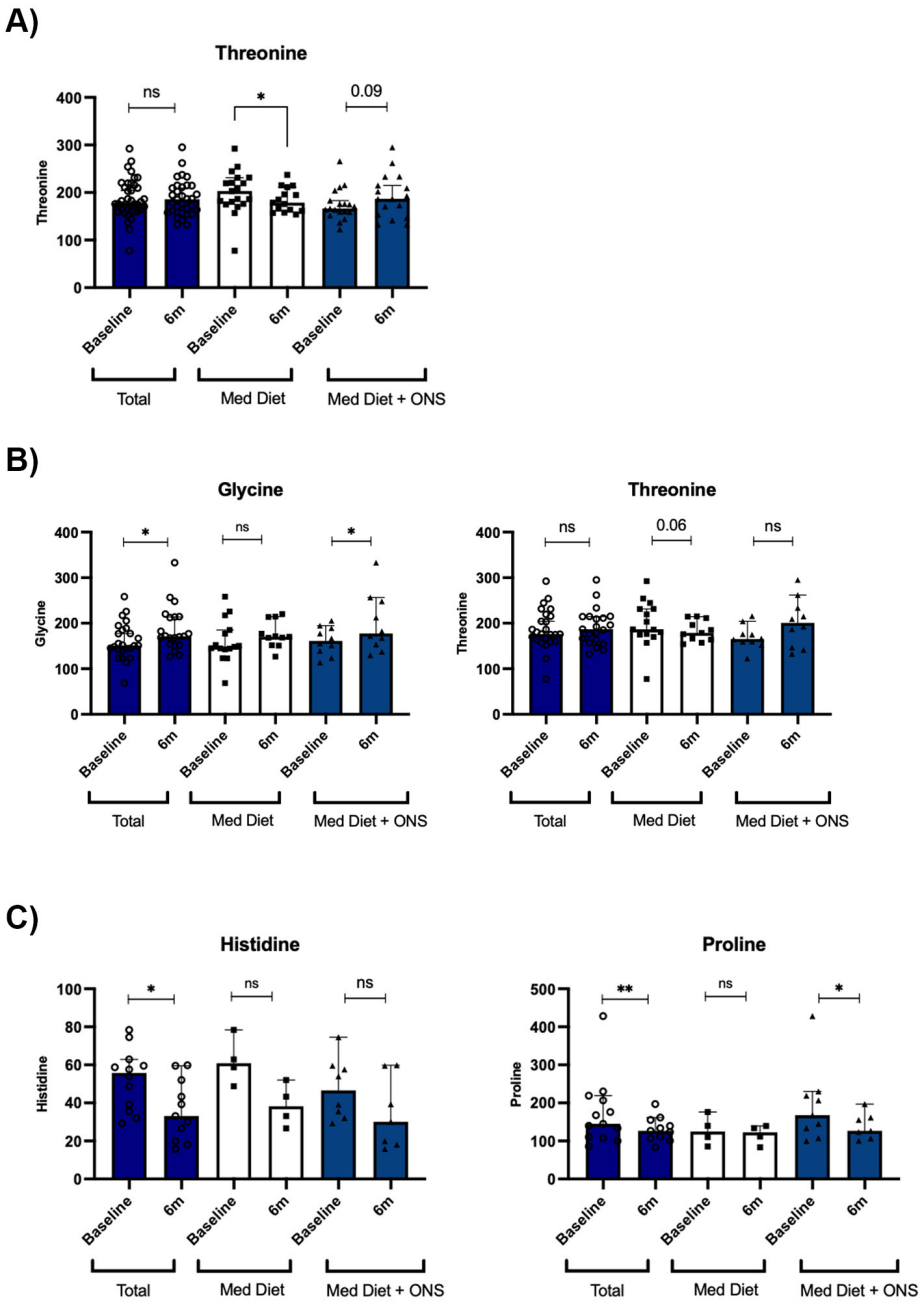
Significant metabolomic changes in amino acid metabolism-related parameters. Analysis of the total study population **(A)** patients with sarcopenia **(B)** and without sarcopenia **(C)**. Legend: ns, non-significant; *p < 0.05; **p < 0.01.

In the subgroup analysis, threonine significantly decreased in the control group (*p* < 0.05), while showing a trend toward increase in the intervention group (*p* = 0.09; Figure 4A).

In patients with sarcopenia, glycine levels significantly increased in the whole cohort (*p* < 0.05), driven by a significant increase in the intervention group (*p* < 0.05). Threonine showed a trend toward decrease in the control group (*p* = 0.06; Figure 4B).

In patients without sarcopenia, histidine significantly decreased in the whole cohort (*p* < 0.05), without group-specific differences. Proline levels significantly decreased in the whole cohort (*p* < 0.01) and specifically in the intervention group (*p* < 0.05; Figure 4C).

### Clinical correlations between cardiac function and metabolomic profile

Baseline NT-proBNP levels were positively correlated with GlycA and GlycB (ρ = 0.440 and 0.552, respectively; *p* < 0.05) and negatively correlated with glycine (ρ = −0.426, *p* < 0.05) and proline (ρ = −0.509, *p* < 0.05).

### Clinical associations with mortality

Mortality was evaluated during the study period. In univariate analyses, baseline creatine and isoleucine levels were significantly associated with mortality (*p* = 0.035 and *p* = 0.006, respectively), while leucine and valine showed non-significant trends (*p* = 0.076 and *p* = 0.062, respectively).

In a multivariable binary logistic regression model adjusted for age and sex, isoleucine remained independently associated with mortality (OR 0.808, 95% CI 0.655–0.996). Additionally, according to a multivariate logistic regression model adjusted for age, sex, the presence of T2DM, baseline NT-proBNP levels, LVEF and BMI, baseline isoleucine and valine remained independently associated with mortality (OR 0.77, 95% CI 0.591–0.9998; OR 0.95, 95% CI 0.907–0.999).

## Discussion

HF is characterized by profound remodeling of both systemic and myocardial metabolism. Circulating low-molecular-weight metabolites reflect these disturbances and provide independent prognostic information (10, 11).

The glycerol fraction in HF reflects circulating levels of proinflammatory glycoproteins, including sgp130 and GlycA (12, 13). Elevated GlycA concentrations have been associated with incident cardiovascular disease, major adverse cardiovascular events, and all-cause mortality. In community-based cohorts, GlycA independently predicts incident HF, particularly HF with preserved ejection fraction, even after adjustment for traditional cardiovascular risk factors and high-sensitivity C-reactive protein (14). Accordingly, a reduction in the glycerol fraction may reflect attenuation of systemic inflammation and IL-6 family signaling, a pattern generally associated with a more favorable prognosis (14). In our study, this pattern was observed in the overall cohort, particularly among patients without sarcopenia who received nutritional counseling.

Energy-related metabolites, such as glucose and pyruvate, reflect the metabolic shift from efficient mitochondrial oxidative phosphorylation toward glycolysis and alternative substrate utilization in the failing myocardium (15). They may also indicate a catabolic or starvation-like state characterized by reduced metabolic reserves, particularly when accompanied by lower circulating concentrations of amino acids and other energy substrates.

Muscle-related metabolic parameters capture the combined effects of cardiorenal dysfunction and skeletal muscle wasting (16, 17). Alterations in tyrosine and other aromatic amino acids have been incorporated into multimarker metabolite scores associated with inflammation, cachexia, and adverse clinical outcomes (18). Branched-chain amino acids (BCAAs; valine, leucine, and isoleucine) accumulate in both plasma and myocardium as a consequence of downregulation of key catabolic enzymes. Impaired BCAA oxidation promotes insulin resistance, mitochondrial dysfunction, and arrhythmogenic remodeling, while elevated circulating BCAA concentrations have been associated with adverse outcomes in observational studies (16). These observations are consistent with the results of our multivariable analysis, which identified isoleucine and valine as independent predictors of mortality.

Metabolites related to amino acid metabolism integrate information on nitrogen handling, anaplerotic flux, and overall nutritional status (16). In our cohort, serum leucine concentrations increased in the intervention group. Experimental studies have shown that leucine-enriched diets promote compensatory hypertrophy, attenuate fibrosis and apoptosis, and favorably modulate oxidative metabolism, thereby improving cardiac structure, function, and survival following acute myocardial infarction (19). Furthermore, animal models suggest that leucine supplementation improves diastolic function and reduces adverse cardiac remodeling (20). The preferential increase observed among patients without sarcopenia may indicate that early nutritional intervention is particularly effective in this subgroup. Similarly, valine concentrations increased in the intervention group. Valine has been recognized as an important regulator of mitochondrial function and cellular bioenergetics (21).

Threonine plays a key role in maintaining muscle mass and supporting cardiovascular function by serving as a substrate for structural proteins and essential metabolic pathways (22). Notably, threonine concentrations tended to increase in the intervention group but decreased significantly in the control group. Consistent with these findings, glycine has been reported to exert both cardioprotective and myoprotective effects, primarily through cytoprotective mechanisms (23, 24). Glycine contributes to cardiovascular protection by enhancing energy metabolism and serving as a precursor for glutathione and creatine, both of which are essential for redox homeostasis, muscle repair, and energy production (25).

Interestingly, proline concentrations decreased during follow-up in patients without sarcopenia, whereas they remained unchanged or increased in those with sarcopenia. Recent evidence suggests that proline dehydrogenase, a key enzyme in proline metabolism, reprograms cardiomyocyte metabolism and protects against pathological cardiac remodeling (26). This finding may therefore reflect enhanced proline catabolism, a mechanism considered protective under conditions of myocardial stress, including hypoxia (27, 28).

Nutritional strategies aimed at modulating these metabolites remain an emerging field and have not yet been incorporated into routine HF management. Small studies evaluating amino acid supplementation have reported improvements in exercise capacity and quality of life; however, robust evidence demonstrating that targeted amino acid interventions can favorably modify prognostic metabolomic profiles or improve hard clinical outcomes remains lacking. Moreover, most available trials have not incorporated comprehensive metabolomic endpoints. Although some studies have reported reductions in inflammatory biomarkers and natriuretic peptides, consistent changes in amino acid or ketone body concentrations have not been demonstrated.

This study has several limitations. First, the relatively small sample size may limit the generalizability of the findings and reduces the statistical power to detect differences between groups. Second, detailed caloric intake data at baseline and study completion were not systematically collected. More importantly, it is not possible to establish a direct causal relationship between any specific component of the ONS formulation (e.g., EPA and DHA enrichment, total caloric content, or protein content) and the observed clinical effects. For ethical reasons, a placebo arm was not included, and the open-label design may have introduced bias during outcome assessment. Nevertheless, future studies comparing different ONS formulations, isolated nutritional interventions (e.g., protein modules, EPA/DHA supplementation, or ONS with alternative protein compositions), or crossover designs could provide valuable mechanistic insights. Given the clinical benefits observed, larger and more comprehensive randomized trials are warranted to confirm these findings and better define the specific contribution of individual ONS components. Although sarcopenia was more prevalent among older participants and may have influenced the univariable analyses, all multivariable models were adjusted for age and sex. The study also has several strengths, including its relatively long duration, contrasting with most nutritional intervention studies in HF, which rarely exceed 3 months, as well as the high adherence to both ONS and the Mediterranean diet.

Overall, current evidence indicates that low-molecular-weight metabolites provide valuable prognostic information in HF. However, definitive evidence demonstrating that nutritional interventions, including omega-3-enriched nutritional support, can consistently normalize these biomarkers and translate into improved clinical outcomes remains limited. This highlights the need for dedicated prospective studies incorporating metabolomics-guided therapeutic strategies.

## Data Availability

The original contributions presented in the study are included in the article/supplementary material, further inquiries can be directed to the corresponding author.

## References

[1] Carbone S Billingsley HE Rodriguez-Miguelez P Kirkman DL Garten R Franco RL . Lean mass abnormalities in heart failure: the role of sarcopenia, sarcopenic obesity, and cachexia. Curr Probl Cardiol. (2020) 45:100417. doi: 10.1016/j.cpcardiol.2019.03.006 31036371 PMC11146283

[2] Yu X Chen Q Xu Lou I. Dietary strategies and nutritional supplements in the management of heart failure: a systematic review. Front Nutr. (2024) 11:1428010. doi: 10.3389/fnut.2024.1428010 39464682 PMC11502353

[3] Ang JC Sun L Foo SR Leow MK Vidal-Puig A Fontana L . Perspectives on whole body and tissue-specific metabolic flexibility and implications in cardiometabolic diseases. Cell Rep Med. (2025) 6:102354. doi: 10.1016/j.xcrm.2025.102354 40961926 PMC12490259

[4] Keller-Ross ML Larson M Johnson BD. Skeletal muscle fatigability in heart failure. Front Physiol. (2019) 10:129. doi: 10.3389/fphys.2019.00129 30846944 PMC6393404

[5] Herrera-Martínez AD Muñoz Jiménez C López Aguilera J Crespin MC Manzano García G Gálvez Moreno MÁ . Mediterranean diet, vitamin D, and hypercaloric, hyperproteic oral supplements for treating sarcopenia in patients with heart failure-a randomized clinical trial. Nutrients (2023) 16:110. doi: 10.3390/nu16010110 38201939 PMC10781070

[6] Fujimoto Y Dotare T Maekawa E Kamiya K Kitai T Kuwahara K . Oral nutritional supplements in older outpatients with heart failure: rationale and design of the ALIMENT-HF trial. ESC Heart Fail. (2024) 11:2379–86. doi: 10.1002/ehf2.14800 38628048 PMC11287343

[7] Billingsley HE Carbone S Driggin E Kitzman DW Hummel SL. Dietary interventions in heart failure with preserved ejection fraction: a scoping review. JACC Adv. (2025) 4:101465. doi: 10.1016/j.jacadv.2024.101465 39801812 PMC11719370

[8] Herrera-Martínez AD Jiménez CM Romo AN Aguilera JL Crespin MC Baena BT . Nutritional support reduces circulating cytokines in patients with heart failure. Nutrients. (2024) 16:1637. doi: 10.3390/nu16111637 38892570 PMC11174422

[9] Ozcariz E Guardiola M Amigó N Rojo-Martínez G Valdés S Rehues P . NMR-based metabolomic profiling identifies inflammation and muscle-related metabolites as predictors of incident type 2 diabetes mellitus beyond glucose: the Di@bet.es study. Diabetes Res Clin Pract. (2023) 202:110772. doi: 10.1016/j.diabres.2023.110772 37301326

[10] Neves LS Saraiva F Ferreira R Leite-Moreira A Barros AS Diaz SO. Metabolomics and cardiovascular risk in patients with heart failure: a systematic review and meta-analysis. Int J Mol Sci. (2024) 25:5693. doi: 10.3390/ijms25115693 38891881 PMC11172189

[11] Fragasso G. Deranged cardiac metabolism and the pathogenesis of heart failure. Card Fail Rev. (2016) 2:8–13. doi: 10.15420/cfr.2016:5:2 28785448 PMC5490933

[12] Gwechenberger M Pacher R Berger R Zorn G Moser P Stanek B . Comparison of soluble glycoprotein 130 and cardiac natriuretic peptides as long-term predictors of heart failure progression. J Heart Lung Transplant. (2005) 24:2190–5. doi: 10.1016/j.healun.2004.10.015 16364870

[13] Askevold ET Nymo S Ueland T Gravning J Wergeland R Kjekshus J . Soluble glycoprotein 130 predicts fatal outcomes in chronic heart failure: analysis from the controlled rosuvastatin multinational trial in heart failure (CORONA). Circ Heart Fail. (2013) 6:91–8. doi: 10.1161/CIRCHEARTFAILURE.112.972653 23230311

[14] An L Liu Q Feng H Bai X Dang Y Li C . Increased glycoprotein acetylation is associated with high cardiac event rates: analysis using coronary computed tomography angiography. Anatol J Cardiol. (2018) 20:152–8. doi: 10.14744/AnatolJCardiol.2018.01058 30152796 PMC6237945

[15] Aubert G Martin OJ Horton JL Lai L Vega RB Leone TC . The failing heart relies on ketone bodies as a fuel. Circulation. (2016) 133:698–705. doi: 10.1161/CIRCULATIONAHA.115.017355 26819376 PMC4766035

[16] Hiraiwa H Okumura T Murohara T. Amino acid profiling to predict prognosis in patients with heart failure: an expert review. ESC Heart Fail. (2023) 10:32–43. doi: 10.1002/ehf2.14222 36300549 PMC9871678

[17] Nakagomi A Kohashi K Morisawa T Kosugi M Endoh I Kusama Y . Nutritional status is associated with inflammation and predicts a poor outcome in patients with chronic heart failure. J Atheroscler Thromb. (2016) 23:713–27. doi: 10.5551/jat.31526 26782970 PMC7399287

[18] Stryeck S Gastrager M Degoricija V Trbušić M Potočnjak I Radulović B . Serum concentrations of citrate, tyrosine, 2- and 3- hydroxybutyrate are associated with increased 3-month mortality in acute heart failure patients. Sci Rep. (2019) 9:6743. doi: 10.1038/s41598-019-42937-w 31043697 PMC6494857

[19] Witham WG Yester KA McGaffin KR. A high leucine diet mitigates cardiac injury and improves survival after acute myocardial infarction. Metabolism. (2013) 62:290–302. doi: 10.1016/j.metabol.2012.07.023 22935555

[20] Alves PKN Schauer A Augstein A Männel A Barthel P Joachim D . Leucine supplementation improves diastolic function in HFpEF by HDAC4 inhibition. Cells (2023) 12:2561. doi: 10.3390/cells12212561 37947639 PMC10648219

[21] Mitrega K Zorniak M Varghese B Lange D Nożynski J Porc M . Beneficial effects of l-leucine and l-valine on arrhythmias, hemodynamics and myocardial morphology in rats. Pharmacol Res. (2011) 64:218–25. doi: 10.1016/j.phrs.2011.04.011 21605982

[22] Tang Q Tan P Ma N Ma X. Physiological functions of threonine in animals: beyond nutrition metabolism. Nutrients. (2021) 13:2592. doi: 10.3390/nu13082592 34444752 PMC8399342

[23] Ramos-Jiménez A Hernández-Torres RP Hernández-Ontiveros DA Ortiz-Ortiz M López-Fregoso RJ Martínez-Sanz JM . An update of the promise of glycine supplementation for enhancing physical performance and recovery. Sports (Basel). (2024) 12:265. doi: 10.3390/sports12100265 39453231 PMC11510825

[24] Martinez BP Gomes IB Oliveira CS Ramos IR Rocha MD Forgiarini Júnior LA . Accuracy of the timed up and go test for predicting sarcopenia in elderly hospitalized patients. Clinics (São Paulo). (2015) 70:369–72. doi: 10.6061/clinics/2015(05)11 26039955 PMC4449469

[25] Dziedzic M Józefczuk E Guzik TJ Siedlinski M. Interplay between plasma glycine and branched-chain amino acids contributes to the development of hypertension and coronary heart disease. Hypertension. (2024) 81:1320–31. doi: 10.1161/HYPERTENSIONAHA.123.22649 38587181 PMC11095885

[26] Lv C Huo R. Association between visceral adiposity index, lipid accumulation product and type 2 diabetes mellitus in US adults with hypertension: a cross-sectional analysis of NHANES from 2005 to 2018. BMC Endocr Disord. (2024) 24:216. doi: 10.1186/s12902-024-01750-x 39407231 PMC11476220

[27] Lv Q Li D Zhao L Yu P Tao Y Zhu Q . Proline metabolic reprogramming modulates cardiac remodeling induced by pressure overload in the heart. Sci Adv. (2024) 10:eadl3549. doi: 10.1126/sciadv.adl3549 38718121 PMC11078183

[28] Wang J Xue Z Lin J Wang Y Ying H Lv Q . Proline improves cardiac remodeling following myocardial infarction and attenuates cardiomyocyte apoptosis via redox regulation. Biochem Pharmacol. (2020) 178:114065. doi: 10.1016/j.bcp.2020.114065 32492448

